# Insights into Perimenopause: A Survey of Perceptions, Opinions on Treatment, and Potential Approaches

**DOI:** 10.3390/women5010004

**Published:** 2025-01-31

**Authors:** Andrea K. Wegrzynowicz, Amanda C. Walls, Myra Godfrey, Amy Beckley

**Affiliations:** 1 Department of Obstetrics and Gynecology, University of Wisconsin-Madison, Madison, WI 53715, USA; 2 MFB Fertility, Boulder, CO 80301, USA; 3 Department of Biomedical Engineering, University of Arkansas, Fayetteville, AR 72701, USA; 4 Jaya Jaya Myra Productions, New York, NY 10010, USA

**Keywords:** perimenopause, menopause, women’s health, hormone replacement therapy (HRT), symptom management, healthcare communication

## Abstract

Perimenopause, the transitional phase leading up to menopause, affects millions of women worldwide, yet it remains poorly understood and under-addressed in healthcare. Despite the availability of treatment options like hormone replacement therapy (HRT) and non-hormonal alternatives, the awareness and utilization of these options vary significantly among women. Here, we conducted a cross-sectional survey with 1000 adults, both men and women, from the United States and Canada. We evaluated the perceived familiarity of participants with the timing, duration, and symptoms of perimenopause, as well as their satisfaction with their treatment options and communication with their healthcare providers. We found that, in general, women and older people were more likely to feel familiar with perimenopause, although the youngest age group surveyed also reported relatively high familiarity. We also found that there is a disconnect between people reporting high familiarity with perimenopause and its symptoms but overall middling and lower familiarity with the age and duration of onset and satisfaction with treatment options. Our results suggest further investigation into where people obtain their information concerning perimenopause, as well as into how knowledge of perimenopause may vary based on demographics.

## 1. Introduction

Perimenopause is defined as the period of time during which a woman’s body transitions towards menopause, or the final menstrual period. The beginning of the perimenopause phase is marked by the onset of menstrual irregularities, and menopause itself is retroactively confirmed after 12 months of amenorrhea. Perimenopause brings about a host of changes within the body, mostly due to the decline in ovarian function, which results in decreased estrogen and progesterone production [1]. The perimenopause phase is reported to last for an average of 4–7 years, though it can last up to 14 years, and the average age of menopause is 51–52 years in the US [1,2].

Each year, approximately 2 million women in the US enter perimenopause [3]. As perimenopause begins, hormones fluctuate, triggering a broad range of unpleasant symptoms such as longer periods of bleeding, anxiety and depression, weight gain, changes in sexual desire, muscle aches, insomnia, and vasomotor dysfunction, which often present as hot flashes and night sweats [1,4]. Most women experience a variety of such symptoms, but it is estimated that at least 20% of women experience symptoms to an extent which significantly impacts their quality of life [5]. Additionally, evidence suggests that the severity of symptoms can vary across different populations of women. One study showed a link between infertility and increased vaginal dryness and decreased libido during menopause [6]. Another study found that African American women were more likely to experience severe vasomotor dysfunction [7]. Ultimately, women experiencing perimenopause and menopause symptoms have reported decreased work productivity, increased activity impairments, and significantly more physician visits compared to nonmenopausal women, indicating a need for treatment [8].

Hormone therapy (HT) is currently the most popular treatment for perimenopause symptoms and has been reported to significantly improve bothersome vasomotor symptoms such as hot flashes and sleep disturbances. Similarly to hormonal birth control, HT can be administered in various forms (i.e., oral pills, vaginal rings, patches, injections), can be given continuously or cyclically, and can contain estrogen, progesterone, or a combination of both [9,10]. Local estrogen treatment may also be prescribed for women who experience genitourinary symptoms such as urinary tract infections. In such cases, local estrogen therapy in the form of vaginal rings, creams, or tablets can help to alleviate symptoms. Currently, HT can be categorized as either synthetic or “bioidentical”, with bioidentical options gaining popularity after several studies reported an alarming association between synthetic HT and an increased risk of cardiovascular disease (CVD) and thromboembolic events [11-16]. Bioidentical hormones are primarily derived from plant products and are intended to mimic a woman’s endogenous hormones. Although there is promising preliminary support for the safety and efficacy of bioidentical hormones, as well as a low incidence of CVD with use, there remains a lack of evidence supporting the superiority of bioidentical HT over synthetic HT to alleviate vasomotor symptoms. Additionally, most studies on bioidentical HT are focused on menopausal and postmenopausal women, so there is limited generalizability to perimenopausal women [17].

Other treatments for perimenopause symptoms include non-hormonal treatments, as well as integrative therapies, lifestyle approaches, and supplementation. Non-hormonal treatments, such as selective serotonin reuptake inhibitors (SSRIs), serotonin–norepinephrine reuptake inhibitors (SNRIs), and gabapentin, have been shown to improve perimenopause symptoms, though few are FDA-approved for this purpose [9]. Evidence-based lifestyle therapies (i.e., the Women’s Wellness Program) have been shown to decrease menopausal symptoms in women through creating sustained positive health behaviors including increased exercise, a quality diet, and improved sleep and stress management [18]. Evidence also suggests that a diet rich in fruits, vegetables, legumes, and whole grains, and including a higher proportion of healthy fats from fish, nuts, and seeds, may help to control vasomotor symptoms [19-21]. Other dietary and botanical supplements have been studied and show varying degrees of impact on symptoms, but further research is needed to elucidate their efficacy [22].

Understanding perimenopause, including what to expect in terms of symptoms and treatment, can play an important role in the ability of women (and partners) to navigate this transitional period. A 2022 UK study surveyed close to one thousand perimenopausal women and found that 63% sought their information from friends, while less than half sought information from a health professional. Evidence across several studies suggests a clear lack a formal education on the biological and psychological impacts of perimenopause and menopause, which subsequently led to worsened symptoms and suboptimal medical care during this time [11,12,23,24]. Prior health conditions and outcomes (for instance, infertility) may also impact perimenopause, and knowledge of this may help women understand what to expect [6].

In this report, we investigate both men and women’s level of familiarity with various aspects of perimenopause and menopause, as well as their feelings towards HTs and their level of satisfaction with care from healthcare providers. Since a lack of information seemed to be a recurring theme in the literature, we also sought to understand women’s attitudes toward the emerging technology of at-home testing. Several companies within the last few years have developed at-home menopause hormone tests in an effort to provide women with more accessible and personalized information. We hypothesized that women may be likely to utilize at-home testing in order to learn more about their individual transition through menopause.

## 2. Results

A total of 1000 people responded to the survey, with 500 respondents identifying as men and 500 respondents identifying as women. While perimenopause primarily affects individuals who identify as women, men were included in this survey for several reasons. One reason was to consider perceptions of perimenopause across society as a whole, not just with those who might experience it. Another reason was that men, especially men partnered with women or individuals going through perimenopause, may also be consumers of perimenopause-related products and health information.

### 2.1. Familiarity with Perimenopause

**Figure 1.**
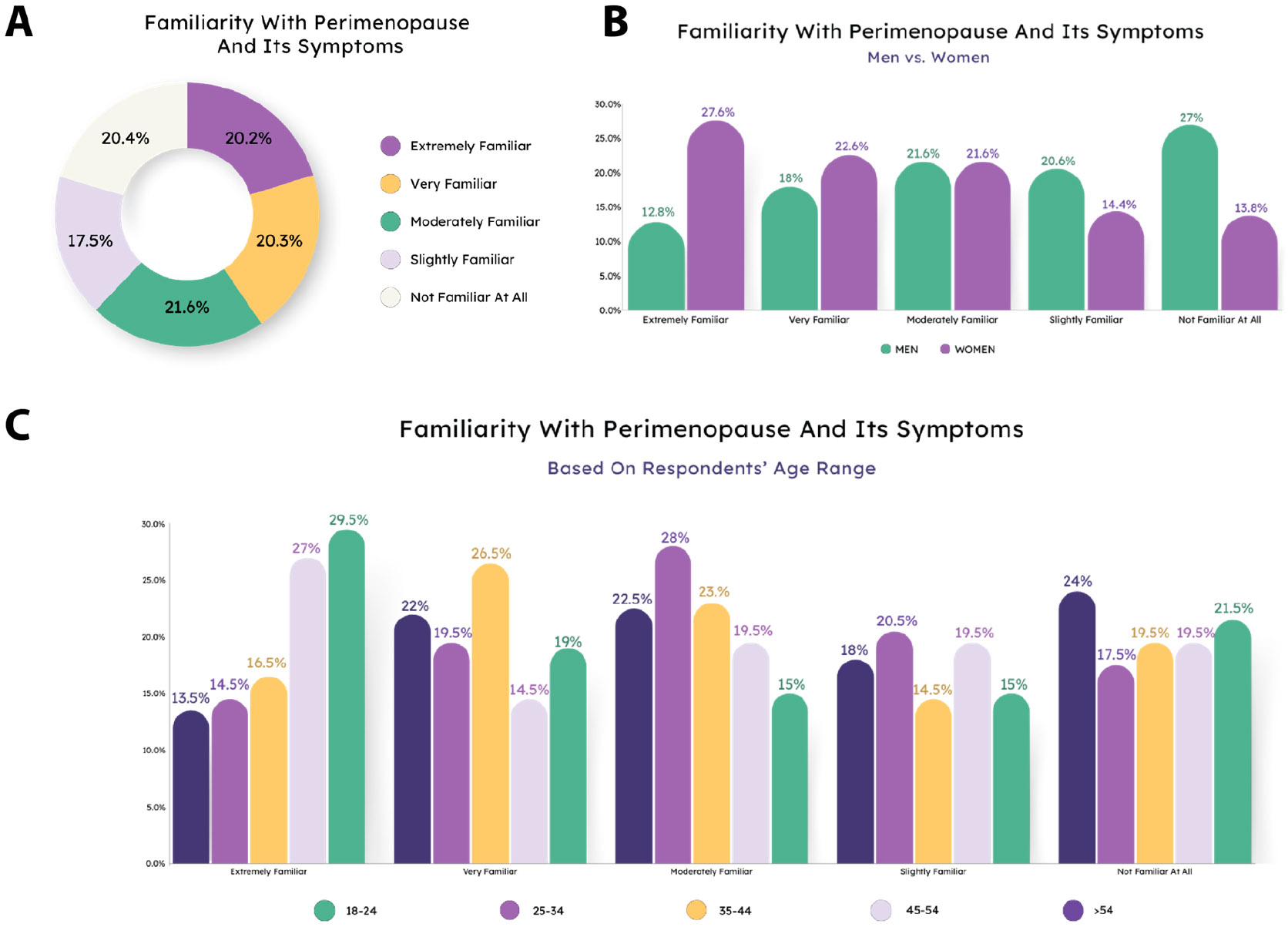
Familiarity with perimenopause and its symptoms depends on gender and age. (**A**) Overall assessment of familiarity with perimenopause. Responses were roughly evenly divided between all familiarity choices. (**B**) Women were more likely to feel familiar with menopause than men. (**C**) Perceived familiarity was dependent upon age.

First, we evaluated how familiar the average person was with perimenopause and its symptoms. The overall results were mostly evenly divided among the five response options (Figure 1A). We hypothesized that women would perceive themselves to be more familiar with perimenopause than men, and this hypothesis was confirmed by our results (X^2^ (df = 4, N = 1000) = 56.559, *p* < 0.0001) (Figure 1B). Age was also a determining factor in perceived familiarity (X^2^ (df = 16, N = 1000) = 43.895, *p* = 0.0002), although the relationship between age and familiarity was not as expected (Figure 1C). The oldest group had the highest percentage of people answering that they were not familiar with menopause symptoms at all, while the youngest and second oldest group were most likely to say they were extremely familiar.

### 2.2. Presumed Age and Duration of Perimenopause

**Figure 2.**
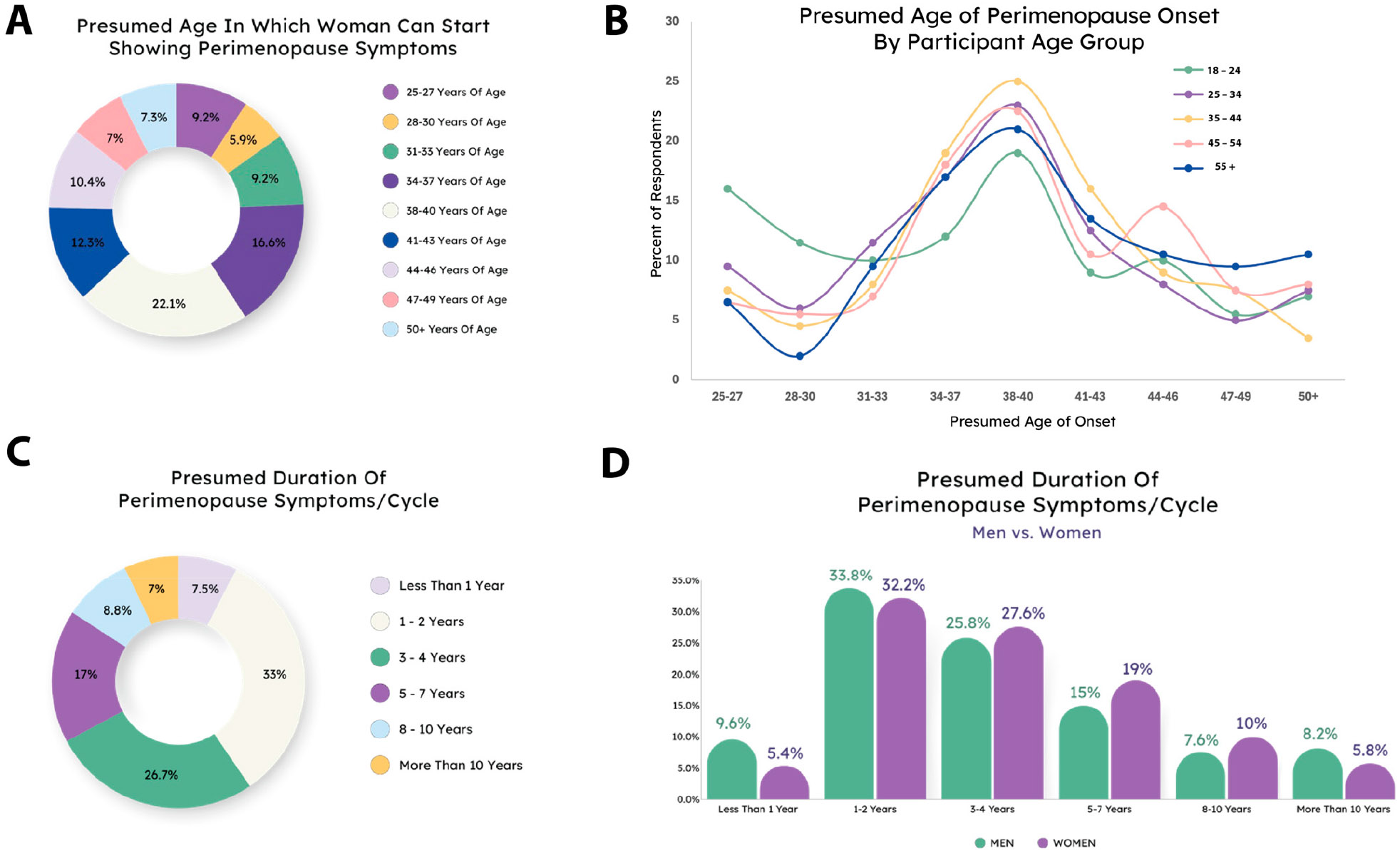
Presumptions of age and duration of perimenopause. (**A**) Presumed age of perimenopause. (**B**) Younger respondents are more likely to believe perimenopause begins earlier than older respondents do. (**C**) Most people presume perimenopause is shorter than it actually is. (**D**) Women are more likely to believe perimenopause has a longer duration than men do.

Next, we wanted to know at what ages people believed perimenopause might begin and how long it lasts. A majority of respondents selected ages ranging from 34 to 43 years old (Figure 2A), which is significantly younger than the generally reported average of 45–54 years [1,4,9,13]. However, some reports indicate that symptoms may begin in some individuals as early as 35 [25], and it is challenging to determine whether this difference between perception and actual age of onset is due to lack of awareness or under-reporting/misunderstanding of perimenopause symptoms. Predicted age of onset did not differ between men and women (X^2^ (df = 8, N = 1000) = 11.08, *p* = 0.1972) (Figure S1A), but respondent age was a determining factor (X^2^ (df = 32, N = 1000) = 58.558, *p* = 0.0028) (Figure 2B). Interestingly, younger respondents were more likely to respond with a younger age and older respondents with an older age. This indicates a potential disconnect between the perception and reality of the onset of perimenopause, particularly in younger respondents.

Similarly, people tended to underestimate the duration of perimenopause. The average duration of symptoms is 4–8 years [26], but the majority of respondents said either 1–2 or 3–4 years (Figure 2C). Women were more likely than men to predict a longer duration (X^2^ (df = 5, N = 1000) = 12.424, *p* = 0.029) (Figure 2D), and older respondents were more likely to predict a longer duration (X^2^ (df = 20, N = 1000) = 53.534, *p* < 0.0001) (Figure S1B).

### 2.3. Familiarity with Treatment Options

**Figure 3.**
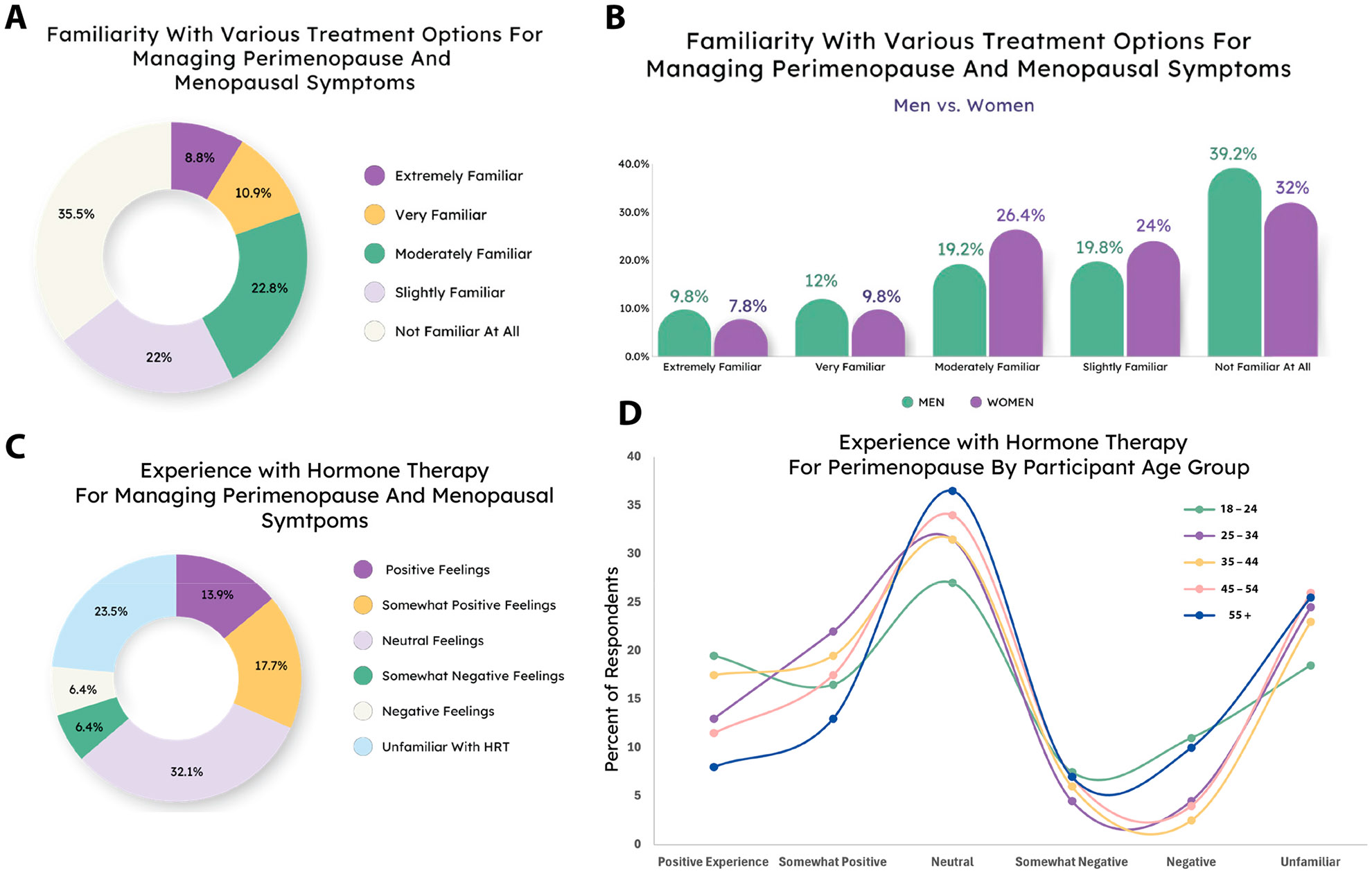
Familiarity with treatments, including HRT. (**A**) Most respondents were not familiar with menopause treatment options. (**B**) Women were slightly more likely to report familiarity with HRT than men. (**C**) The majority of respondents felt neutral or were unfamiliar with HRT. (**D**) Experience with HRT is dependent upon the age of respondent.

Next, we investigated familiarity with treatment options and experience with hormone replacement therapy, the most effective and popular option for symptom management [27]. Most people surveyed were not very familiar with available treatment options, which might be expected given the ages included in the survey (Figure 3A). However, we did not find statistical dependence between age and familiarity (X^2^ (df = 16, N = 1000) = 23.459, *p* = 0.102) (Figure S2A). Women were, however, more likely to report that they were familiar with treatment options than men were (X^2^ (df = 4, N = 1000) = 13.585, *p* = 0.0087) (Figure 3B).

We then asked specifically about hormone replacement therapy (HRT). The majority of respondents were either unfamiliar or had neutral feelings about HRT, again consistent with expectations given that most of our survey population has likely never used HRT for perimenopause management (Figure 3C). However, further investigation suggests that the populations most likely to have used HRT are no more likely (or even less likely) to report positive feelings about it than those who have not, based on our data. There was no statistical difference in the distribution of responses based on gender (X^2^ (df = 5, N = 1000) = 8.3169, *p* = 0.1396) (Figure S2B), and while results did vary significantly based on age of respondent (X^2^ (df = 20, N = 1000) = 43.778, *p* = 0.0016), the two oldest age groups were the least likely to report a positive experience (Figure 3D).

### 2.4. Use of At-Home Tests for Perimenopause

**Figure 4.**
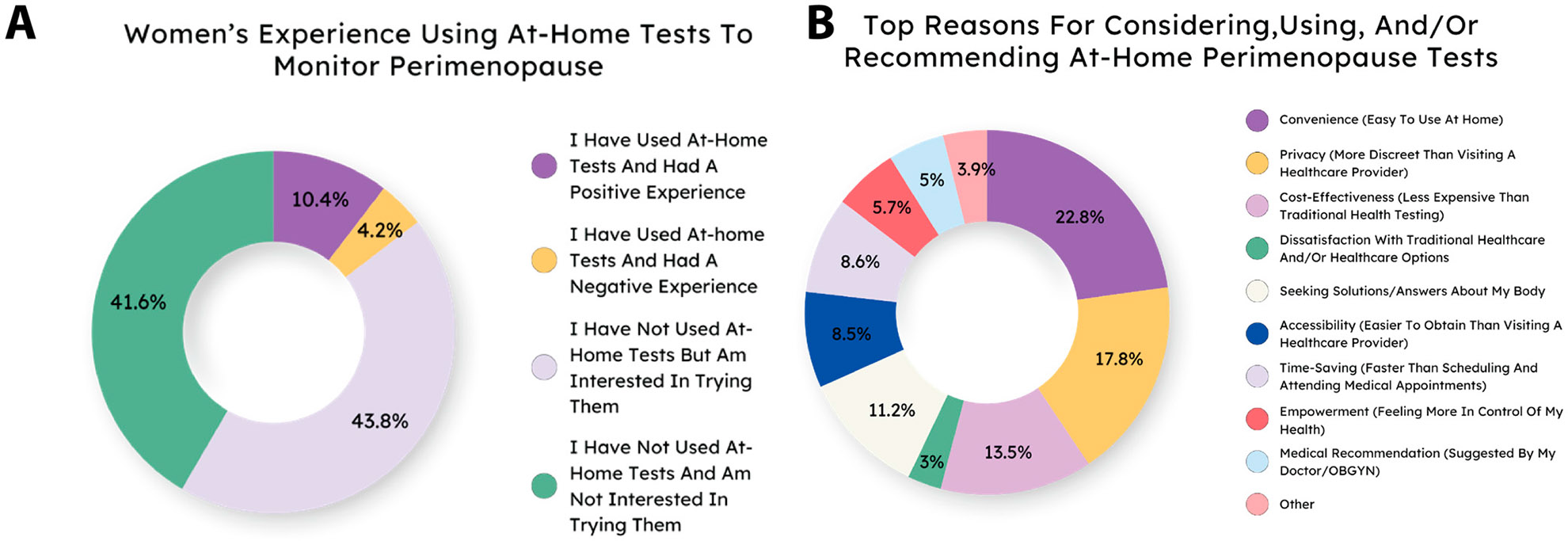
Experiences using at-home tests. (**A**) The majority of women have not used at-home tests for perimenopause, but many are interested. (**B**) Reasons for using or recommending at-home tests vary.

At-home fertility and similar hormone tests are useful and increasingly popular for assisting with screening for hormonal conditions and fertility status [28-31]. Increasingly, at-home tests for perimenopause and related symptoms are becoming available, but they have not been thoroughly evaluated either for ease of use or clinical necessity. We asked respondents whether they had used at-home tests for perimenopause. The majority of respondents had not, but a plurality said they would be interested in trying them, indicating potential consumer/patient demand for at-home testing (Figure S3A). Removing men did not change the results (Figure 4A and Figure S3B,C). When asked about reasons for using or recommending at-home perimenopause tests, women had a range of answers, but the most popular were convenience, privacy, and cost (Figure 4B). Results were typically consistent across age group and gender, with notable exceptions being that women were more likely to seek tests for answers about their body and the 54+ age group was the most likely to use the tests based on a medical recommendation (Figure S4).

### 2.5. Satisfaction Regarding Healthcare Communication Surrounding Menopausal States

**Figure 5.**
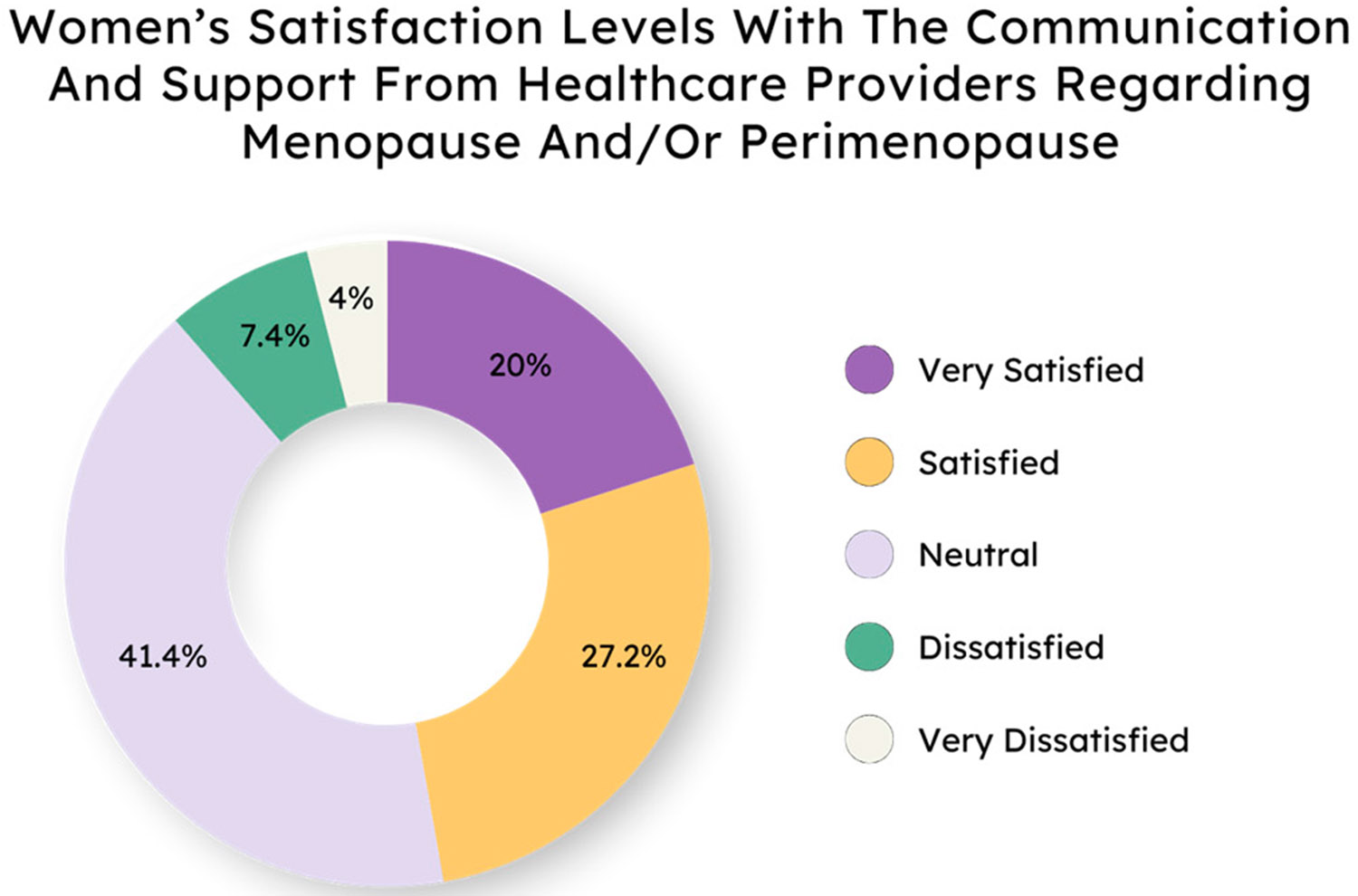
Most women are satisfied or feel neutral about communication and support from their healthcare providers.

Finally, we wanted to assess whether people were satisfied with the communication received from their health care providers with regard to menopause. Most people (Figure S5A) and women specifically (Figure 5) were satisfied or neutral regarding their communication and support from their healthcare practitioners about perimenopause, regardless of age (Figure S5B). This was interesting and suggests further follow-up to determine where people are most likely to receive their information about menopause and perimenopause.

## 3. Discussion

Despite 75 million women in the U.S. currently living in a menopausal state, there is a lack of knowledge about perimenopause, including when it begins, how long it lasts, and the available treatment options. The collective data from this survey indicate that there may be a lack in awareness and understanding of perimenopause and its management within the general population. Of note, we found that nearly one-third of respondents had little or no familiarity with perimenopause or menopause, and over half had little or no familiarity with available treatments. This represents a gap in general knowledge of perimenopause and menopause and indicates that many women may be unprepared to effectively manage their symptoms during this critical transition period.

The lack of general knowledge on perimenopause and menopause is further reflected in the misconceptions about the onset and duration of perimenopause, though this could be due in part to the fact that the literature, healthcare providers, and online sources all suggest different timeframes. Many studies suggest that most women have begun perimenopause by age 45–47, and the average age of menopause is 51–52 in the US [1,2,25,32]. This suggests that the perimenopause period can last nearly seven years in some cases. It is important to note that perimenopause can be categorized as either early or late perimenopause, and the stage one chooses to define perimenopause onset will alter the duration. Early perimenopause refers to the onset of occasional menstrual irregularity, while late perimenopause is defined as 60 or more days without a period. Reproductive history, age of onset, and other health and lifestyle factors can all affect the duration and symptoms of early and late stages of perimenopause [32]. Evidence suggests that most women will only experience vasomotor symptoms during late perimenopause, which typically lasts only 1–2 years, but women who transition to perimenopause earlier may spend a much longer time in the early stage [25]. Our survey results suggest that many women perceive perimenopause as having a longer duration than only the late perimenopause stage. Approximately 40% of our survey respondents believe that perimenopause lasts 2 years or less, while nearly 44% believe it lasts 3–7 years. This divide in perceptions suggests a lack of unified understanding on the onset of perimenopause and may be due in part to varying experiences with early versus late perimenopause symptoms and duration.

We also report that there seems to be a deficiency in widespread awareness and use of treatment options for perimenopause and menopause symptoms. Less than half of women felt at least moderately familiar with available treatments. Additionally, 25% of respondents were unaware of HT, a common treatment for managing menopause symptoms, albeit less common for perimenopause. It is important to note that other types of treatments, such as diet and lifestyle changes, can have a positive effect on menopausal symptoms, and more women may be turning towards these non-hormonal treatments. Our survey did not specifically ask respondents about their familiarity with treatments outside of HT, but future studies could aim to further unpack knowledge and attitudes towards various types of menopause treatments. Future studies could also specifically investigate how experiences and treatment usage vary within different age groups for women specifically.

While a significant number of women reported feeling neutral—neither satisfied nor dissatisfied—about the support they receive from healthcare providers regarding menopause and perimenopause, future directions could include investigating what information is provided to patients. This neutrality suggests a need for more proactive and informative communication from healthcare professionals to better support women during this transition.

At-home testing for menopause is a technology that has only emerged within the last few years, which likely explains why 80.6% of respondents have never used an at-home test to monitor their symptoms. However, over 60% of respondents have tried or are interested in trying an at-home test, with convenience, privacy, and cost-effectiveness being the main motivations for their interest. These results suggest that women likely want to increase their knowledge of menopause and their personal transition through this period, and that at-home testing may be a well-utilized tool for helping women due to its convenience and accessibility.

One limitation of our study was the inability to collect specific demographic data, and it is important to understand how providers may need to treat the needs of specific perimenopausal populations differently. This may include the use of validated tools and surveys to assess perimenopausal status even for younger women, as well as the development of future validated surveys to understand how perceptions of perimenopause may vary with specific demographics. Another limitation was that, due to the survey platform used, data were collected in an aggregated manner, preventing the calculation of Cronbach’s alpha or similar measures. Our sample was also limited to English-speaking smartphone users, although this does include the vast majority of the US and Canadian populations. Future studies should include demographic data and varied sampling methods to assess how race, income, education level, language spoken, and other demographic factors impact familiarity with perimenopause and its symptoms.

The findings of this survey clearly indicate that more extensive education and communication efforts are necessary to bridge the gap between the reality of perimenopause and the widespread lack of recognition and understanding it currently receives. We believe this change can first be initiated at the healthcare provider level, as many providers lack the appropriate knowledge to manage patients in the perimenopause and menopause stage. Providers can seek more formal training through CME credits and post-graduate education. Additionally, more educational resources and courses for providers should be developed by professional associations, such as the American College of Obstetrics and Gynecology (ACOG). For translation of information from provider to patient, providers should strive to educate women nearing perimenopause about symptom expectations, testing options, and all available treatments. In-office literature and social media pages could be utilized as an avenue for information-sharing as well. Empowering women with accurate information and accessible treatment options will not only improve their quality of life but also reduce the stigma associated with this natural stage of life.

## 4. Methods

To understand how familiarity with perimenopause, its symptoms, and potential treatments are distributed across ages and genders, we surveyed 1000 people from the United States and Canada. This survey was conducted using an online survey platform, Pollfish, which provided aggregated/deidentified results. The survey platform advertised the survey with Random Device Engagement through third-party apps [33]. Random users of mobile apps were presented with the opportunity to take the survey to receive non-monetary incentives through the apps. These users then specifically opted in and consented to the survey through the survey platform. Survey results were capped to create an even gender and age distribution throughout the categories chosen—that is, 500 women and 500 men were surveyed, and there were 100 participants of each gender per age group (18–24, 25–34, 35–44, 45–54, 55+). N = 1000 was chosen to meet a minimum threshold of 100 participants within each smaller group, as well as to meet cost and feasibility requirements. Only the age and gender of participants were collected by the survey platform. There were no exclusion criteria for this survey. Gender identity was self-reported.

The survey consisted of eight questions with categorical responses (Supplementary S1). The questions were developed to assess overall familiarity with perimenopause, its symptoms, and potential treatments towards the aims of developing future tools, products, and recommendations for clinicians. Results were analyzed by frequency, gender, and age category. Chi-square tests of independence were chosen to assess the significance and relationship of age and gender to response/outcome. In each case, the null hypothesis was that groups of different age and/or gender would answer with the same distribution. Chi-square tests were used to support or reject the null hypothesis, with rejection indicating that age and/or gender groups chose responses with significantly different frequencies. All participants answered every question. Statistical analyses were carried out using base R.

## Supplementary Material

Supplementary Materials

## Data Availability

The data presented in this study are available on request from the corresponding author. Confidentiality and anonymity were strictly maintained, with no personal identifying information collected in any way to protect participants’ privacy. The data were stored securely and only accessible to authorized researchers involved in the study. Any reporting of findings was performed in a way that aggregated data and did not identify individual respondents.
