# Supplementary material for: Insights into Perimenopause: A Survey of Perceptions, Opinions on Treatment, and Potential Approaches
Source: Women (Basel). Author manuscript; Available in PMC 2025 Apr 22. (PMC12014197; doi:10.3390/women5010004)
Supplement: Supplementary Materials [file NIHMS2071635-supplement-Supplementary_Materials.pdf]

A

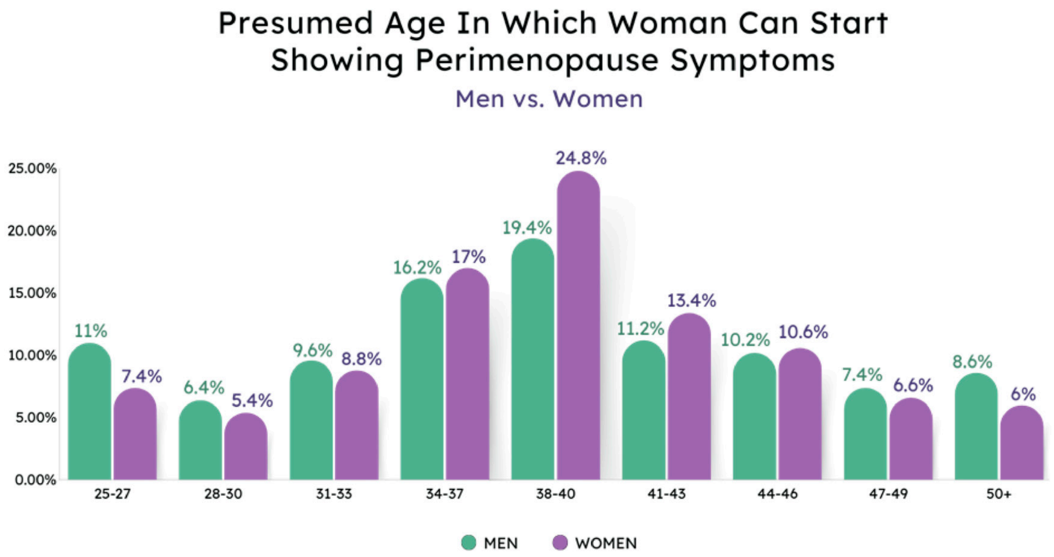

B

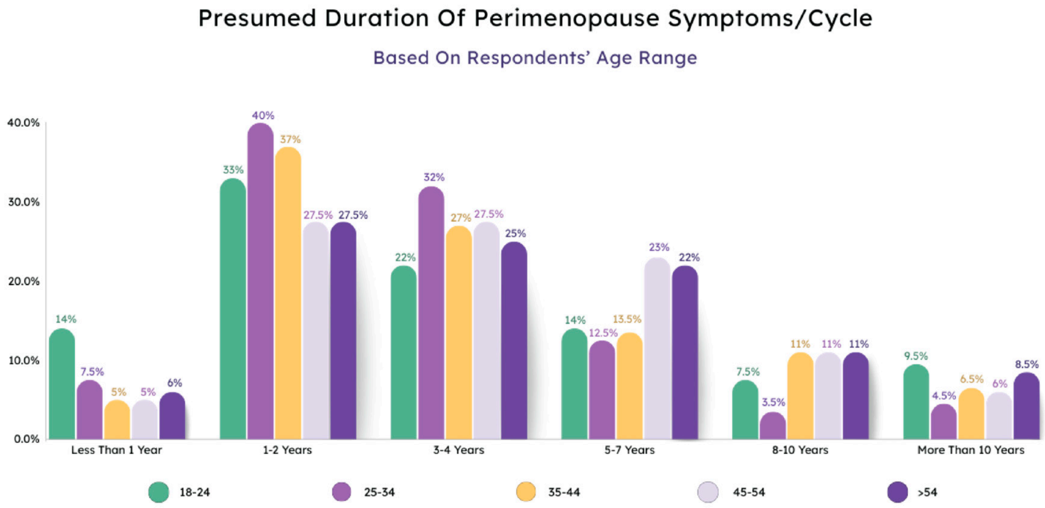

Figure S1. Presumed Duration of Perimenopause Symptoms. (**A**) Men and women overall do not respond differently to the presumed age of perimenopause onset. (**B**) younger age groups are more likely to believe menopause is of a shorter duration.

A

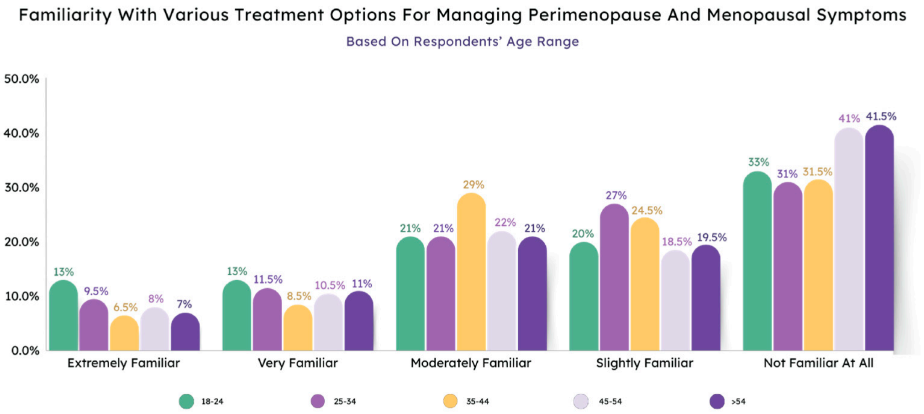

B

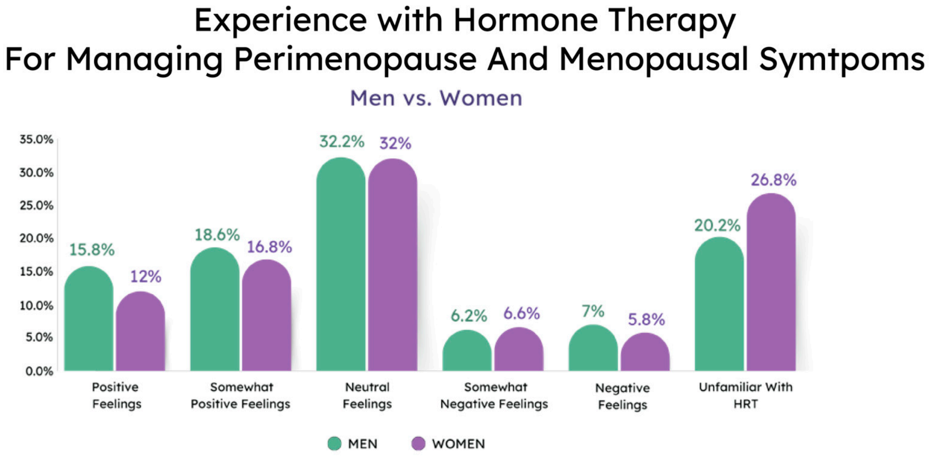

Figure S2. Familiarity and Experience with Menopausal Treatment Options. (**A**) Most age ranges had consistent familiarity with management options for perimenopause. (**B**) Gender-specific results were similar for experiences with HT.

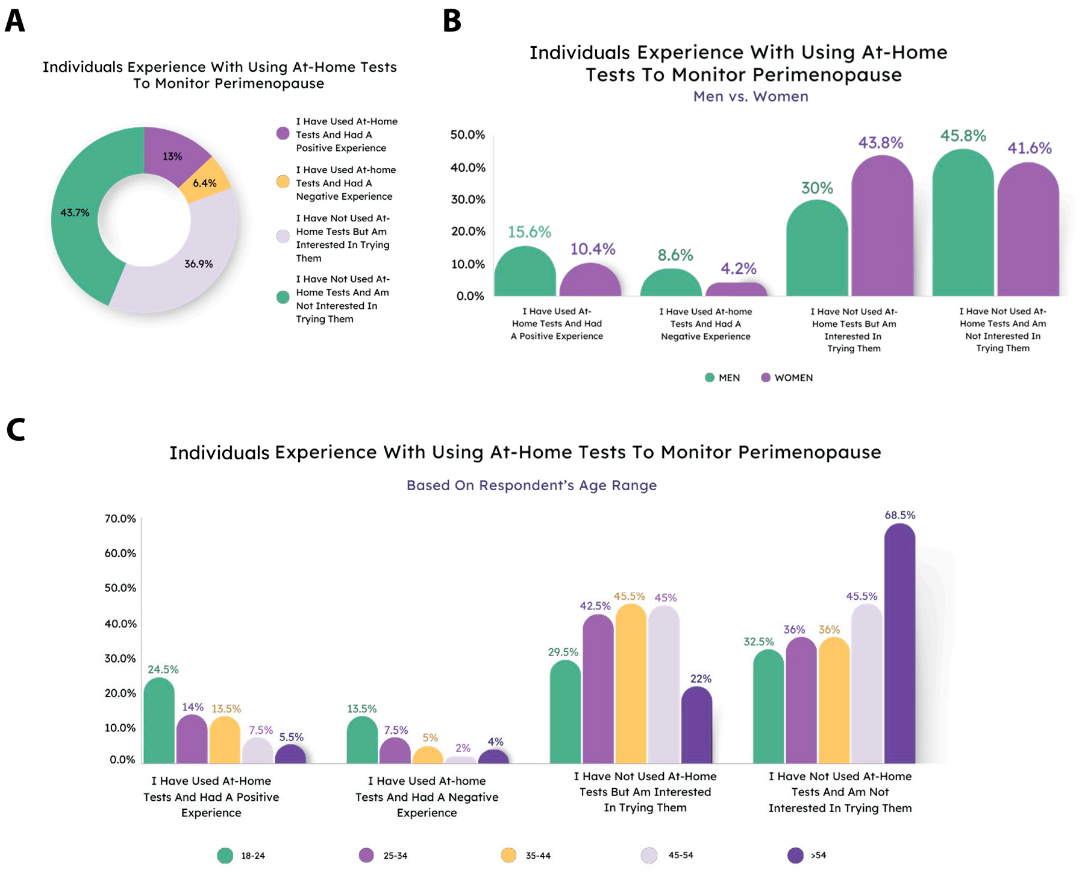

Figure S3. Experiences using At-Home Tests to Monitor Perimenopause. (**A**) Individual results for experiences using at-home tests, including both genders. (**B**) Gender-specific results for experience using at-home tests. C) Age-group specific, mixed-gender results for experiences using at-home tests.

Supplementary S1

Survey Questions

1. How familiar are you with perimenopause and its symptoms? (Perimenopause is the transition period before menopause when a woman's hormone levels and menstrual cycles start to change).
    1. Extremely familiar
    2. Very familiar
    3. Moderately familiar
    4. Slightly familiar
    5. Not familiar at all
2. What is the earliest age you believe a woman can start showing perimenopause symptoms (Perimenopause is the transition period before menopause when a woman's hormone levels and menstrual cycles start to change)?
    1. 25–27
    2. 28–30
    3. 31–33
    4. 34–37
    5. 38–40

6. 41–43
7. 44–46
8. 47–49
9. 50 or older

3. How many years do you think the transition to menopause (otherwise known as perimenopause) typically lasts?
    1. Less than 1 year
    2. 1–2 years
    3. 3–4 years
    4. 5–7 years
    5. 8–10 years
    6. More than 10 years
4. How familiar are you with the treatment options that are available for managing perimenopause and menopausal symptoms?
    1. Extremely familiar
    2. Very familiar
    3. Moderately familiar
    4. Slightly familiar
    5. Not familiar at all
5. What has been your experience with using at-home tests to monitor perimenopause?
    1. I have used at-home tests and had a positive experience
    2. I have used at-home tests and had a negative experience
    3. I have not used at-home tests but am interested in trying them
    4. I have not used at-home tests and am not interested in them
6. What is or would be your primary reason for considering or using at-home perimenopause tests? (Select all that apply).
    1. Convenience (easy to use at home)
    2. Privacy (more discreet than visiting a healthcare provider)
    3. Cost-effectiveness (less expensive than traditional health testing)
    4. Dissatisfaction with traditional healthcare (not satisfied with current healthcare options)
    5. Seeking solutions/answers about my body (wanting to understand my body better)
    6. Accessibility (easier to obtain than visiting a healthcare provider)
    7. Time-saving (faster than scheduling and attending medical appointments)
    8. Empowerment (feeling more in control of my health)
    9. Preference for home testing (prefer testing at home over clinical settings)
    10. Medical recommendation (suggested by my doctor/OBGYN)
    11. Other

7. How satisfied are you with the communication and support from your healthcare provider regarding menopause or perimenopause?
    1. Very satisfied
    2. Satisfied
    3. Neutral
    4. Dissatisfied
    5. Very dissatisfied
8. What are your thoughts on hormone replacement therapy (HRT) for managing perimenopause and menopausal symptoms?
    1. I have positive feelings about HRT
    2. I have somewhat positive feelings about HRT
    3. I have neutral feelings about HRT
    4. I have somewhat negative feelings about HRT
    5. I have negative feelings about HRT
    6. I do not know anything about HRT

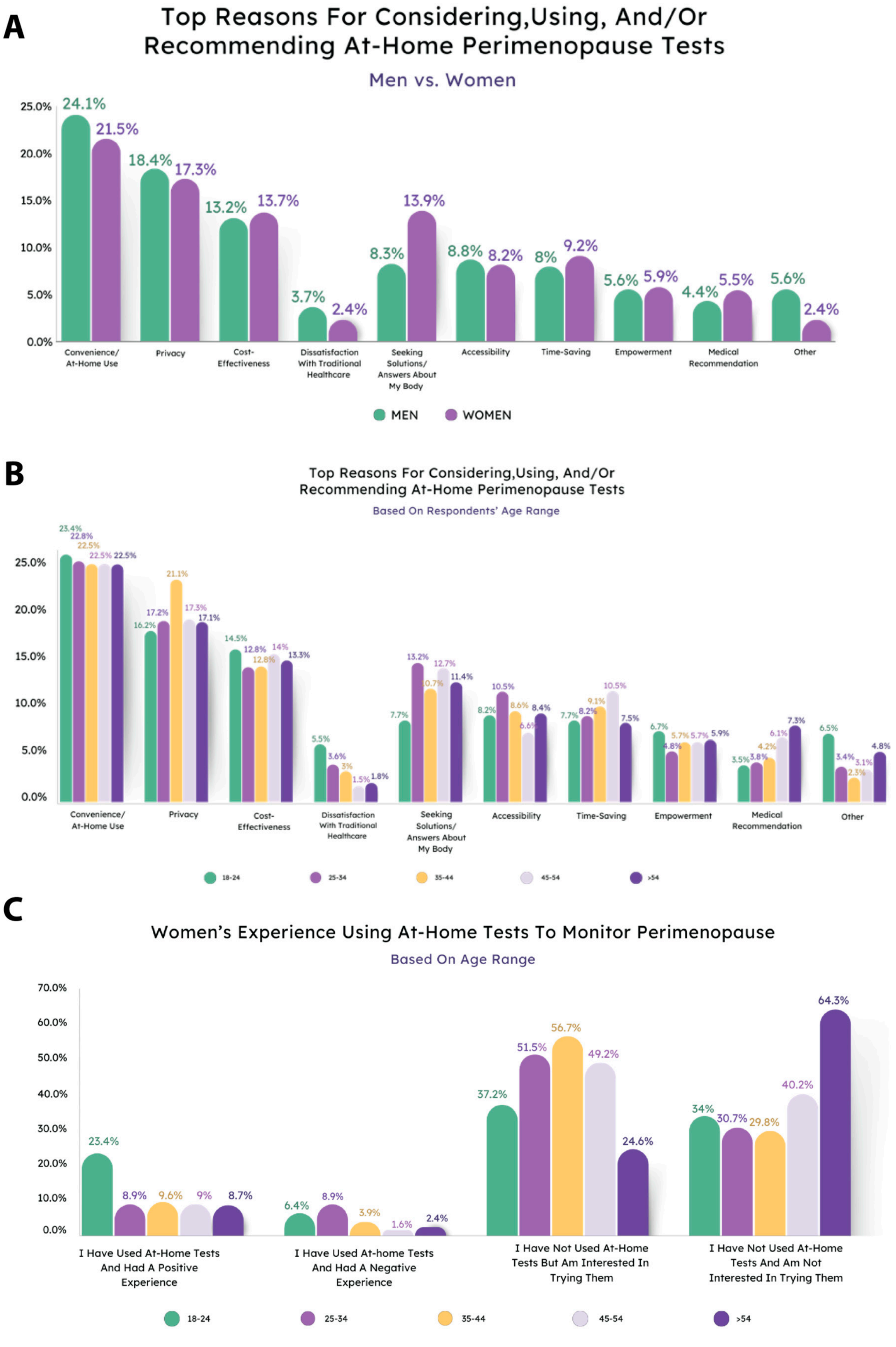

Figure S4. Reasons for Consideration and Experience of Using At-Home Tests. (**A**,**B**) Gender and age-group specific answers for the top reasons for

considering, using, or recommending at-home tests. (**C**) Distribution of experiences and desires of using at-home tests by age group.

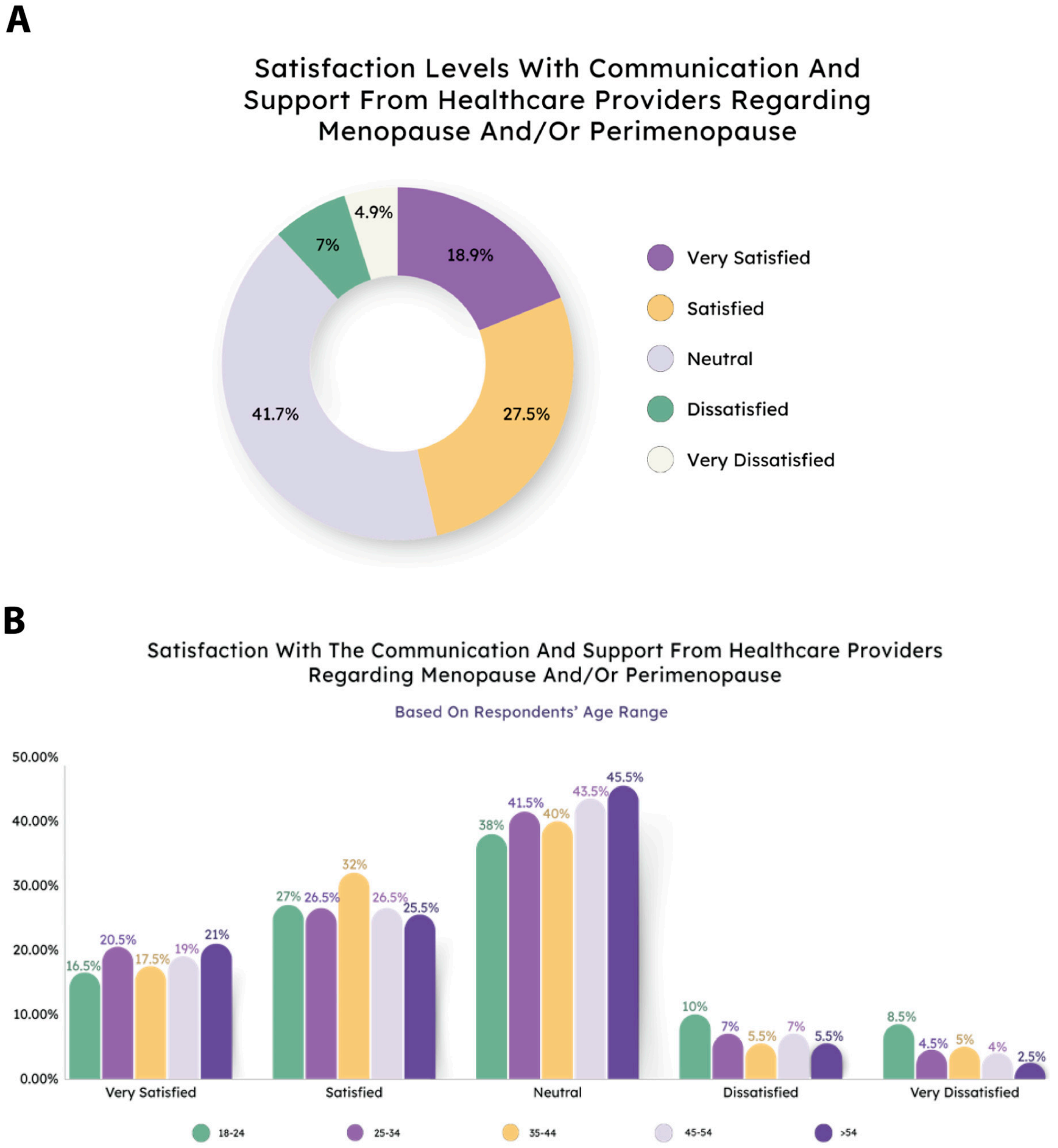

Figure S5. Satisfaction with Communication from Healthcare Providers. (**A**) Overall satisfaction levels and (**B**) age-group specific satisfaction levels with communication and support from healthcare providers regarding perimenopause and/or menopause.
